# ^123^I-FP-CIT SPECT validation of nigro-putaminal MRI tractography in dementia with Lewy bodies

**DOI:** 10.1186/s41747-020-00153-6

**Published:** 2020-05-04

**Authors:** Matteo Pardini, Flavio Nobili, Dario Arnaldi, Silvia Morbelli, Matteo Bauckneht, Roberto Rissotto, Carlo Serrati, Gianluca Serafini, Caterina Lapucci, Lucio Ghio, Mario Amore, Davide Massucco, Davide Sassos, Laura Bonzano, Giovanni Luigi Mancardi, Luca Roccatagliata, the Disease Management Team on Dementia of the IRCCS Ospedale Policlinico San Martino, Genoa

**Affiliations:** 1 Department of Neuroscience, Rehabilitation, Ophthalmology, Genetics, Maternal and Child Health (DINOGMI), University of Genoa, Genoa, Italy; 2 Ospedale Policlinico San Martino IRCCS, Genoa, Italy; 3 Department of Health Sciences (DISSAL), University of Genoa, Genoa, Italy; 4 Nuclear Medicine Unit, IRCCS Ospedale Policlinico San Martino, Genoa, Italy; 5 Psychiatry Branch, Galliera Hospital, Genoa, Italy; 6 Neurology Unit, Ospedale Antero Micone, Genoa, Italy; 7 Department of Neuroradiology, Ospedale Policlinico San Martino IRCCS, Genoa, Italy

**Keywords:** Alzheimer disease, Diffusion tensor imaging, Ioflupane, Magnetic resonance imaging, Tomography (emission-computed, single-photon)

## Abstract

**Background:**

Assessment of nigrostriatal degeneration is a key element to discriminate between dementia with Lewy bodies (DLB) and Alzheimer disease (AD), and it is often evaluated using ioflupane (^123^I-FP-CIT) single-photon emission computed tomography (SPECT). Given the limited availability of ^123^I-FP-CIT SPECT, we evaluated if a mask-based approach to nigroputaminal magnetic resonance imaging (MRI) diffusion-weighted tractography could be able to capture microstructural changes reflecting nigroputaminal degeneration in DLB.

**Methods:**

A nigroputaminal bundle mask was delineated on 12 healthy volunteers (HV) and applied to MRI diffusion-weighted data of 18 subjects with DLB, 21 subjects with AD and another group of 12 HV. The correlation between nigroputaminal fractional anisotropy (FA) values and ^123^I-FP-CIT SPECT findings was investigated. Shapiro-Wilk, ANOVA, ANCOVA, and parametric correlation statistics as well as receiver operating characteristic (ROC) analysis were used.

**Results:**

DLB patients showed a higher nigroputaminal FA values compared with both AD and HV-controls groups (*p* = 0.001 for both comparisons), while no difference was observed between HV-controls and AD groups (*p* = 0.450); at ROC analysis, the area under the curve for the discriminating DLB and AD subjects was 0.820; FA values correlated with ^123^I-FP-CIT values (on the left, *r* = -0.670; on the right, *r* = -720). No significant differences were observed for the FA of the corticospinal tract across the three groups (*p* = 0.740).

**Conclusions:**

In DLB, nigroputaminal degeneration could be reliably assessed on MRI diffusion scans using a mask of nigroputaminal bundle trajectory. Nigroputaminal FA in DLB patients correlated with ^123^I-FP-CIT values data may allow to differentiate these patients from AD patients and HV-controls.

## Key points

- Distinction of dementia with Lewy bodies (DLB) from the Alzheimer disease is the most common problem during DLB work-up.
- Magnetic resonance imaging diffusion-weighted techniques studying the nigrostriatal tracts in the Parkinson disease revealed conflicting results.
- ^123^I-FP-CIT single-photon emission computed tomography is a currently accepted indicative biomarker for DLB diagnosis.
- Diffusion-weighted MRI may capture nigroputaminal microstructural changes in DLB patients.

## Background

Dementia with Lewy bodies (DLB) represents the second most frequent dementing illness in subjects aged 65 years and above [1]. While DLB differential diagnosis is quite broad, its distinction from Alzheimer disease (AD) is the most common problem during DLB work-up [2, 3]. To help in this differential diagnosis, dopamine transporter (DAT) single-photon emission computed tomography (SPECT) with ^123^I-FP-CIT is to date the best standardised technique [3, 4], and it is included in current DLB diagnostic criteria as an indicative biomarker [1]. The rationale of this approach lies on the degeneration of the nigrostriatal pathway in DLB but not in AD which ^123^I-FP-CIT-SPECT captures as a reduction of radiopharmaceutical specific binding ratio.

Fibre tractography represents an analysis approach to diffusion-weighted magnetic resonance imaging (MRI) data used to identify specific white matter tracts [5]. Calculation of diffusion-related metrics within the identified tracts can then assess tissue microstructure and could be able to capture nigrostriatal degeneration. Using tractography, however, the identification of the nigrostriatal pathway is not straightforward, as nigrostriatal fibres are mainly unmyelinated and their trajectories are curved [6]. Indeed, the use of diffusion-weighted MRI techniques to study the nigrostriatal tracts in healthy controls and in movement disorders such as the Parkinson disease (PD) has revealed conflicting results. For example, nigrostriatal fractional anisotropy (FA) has been reported to be increased [7], decreased [8], or unchanged [9] in PD patients compared to controls. Scant evidence, moreover, is available regarding the use of tractography to study the nigrostriatal pathway in DLB.

Here, we decided to evaluate nigroputaminal (NP) white matter integrity in DLB patients compared to AD and controls and to take advantage of the inclusion of ^123^I-FP-CIT-SPECT imaging in the DLB work-up to use it to validate NP tractography results.

## Methods

### DLB and AD patients

In this study, we recruited 18 subjects with a diagnosis of DLB, aged 77.0 ± 1.1 years (mean ± standard deviation), 8 males and 9 females, with a Mini-Mental State Examination (MMSE) score of 25.7 ± 2.1 (Table 1) according to current criteria [1] and 21 subjects with a diagnosis of mild AD dementia, aged 76.4 ± 1.0, 9 males and 12 females, with a MMSE score of 25.9 ± 2.3 (Table 1) according to the National Institute on Aging-Alzheimer’s Association criteria [10].

**Table 1:** Demographic and neuropsychological features of patients and controls

	**Alzheimer disease**	**Dementia with Lewy bodies**	**HV-controls**	**HV-tractography**
**Age (years)**	76.4 ± 1.0	77.0 ± 1.1	75.4 ± 1.6	76.1 ± 1.5
**Sex (males/females)**	9/12	8/9	6/6	5/7
**MMSE (raw score)**	25.9 ± 2.3	25.7 ± 2.1	29.3 ± 1.0	29.0 ± 1.1
**CDR-SB**	4.8 ± 1.3	4.5 ± 1.4	**−**	**−**
**IADL lost**	1.2 ± 0.4	1.1 ± 0.6	**−**	**−**

Data are reported as mean ± standard deviation. *HV* Healthy volunteers; *CDR-SB* Clinical dementia rating scale, sum of boxes; *IADL* Instrumental activities daily living

All subjects were enrolled from a tertiary referral centre among those presenting with a differential diagnosis between DLB and AD and who underwent clinical and neuropsychological testing, brain MRI and ^123^I-FP-CIT-SPECT imaging during their work-up. Moreover, all subjects underwent a brain ^18^F-fluorodeoxyglucose (^18^F-FDG) positron emission tomography (PET) scan during their clinical work-up. Subjects presenting with a normal ^18^F-FDG-PET scan or an anterior pattern of hypo-metabolism were considered not to be eligible, while all subjects presented with a posterior pattern of hypo-metabolism felt to be possibly affected with AD or DLB. Details on the ^18^F-FDG-PET acquisition were reported elsewhere [11]. To be included in the study, subjects needed to present clearly positive or negative ^123^I-FP-CIT-SPECT scan (*i.e.*, evidence of nigrostriatal degeneration for all DLB subjects and for none of the AD subjects based on normative data [12]) performed no later than 6 months after the first access to our centre and to have a clinical follow-up of at least 3 years after the ^123^I-FP-CIT-SPECT scan confirming the baseline clinical diagnosis.

To the purpose of this study, only diffusion MRI and ^123^I-FP-CIT-SPECT data were taken into account. Subjects with neurological, psychiatric or major medical co-morbidities, as well as with a MMSE total score of less than 23, were considered not eligible for the study. MMSE, clinical dementia rating scale sum of boxes [13] and instrumental activities of daily living lost [14] are reported in Table 1. Furthermore, the presence of a vascular burden was excluded by the use of the Fazekas scale [moderate/severe] as an exclusion criterion.

### Healthy controls

We recruited two groups, each of them composed of 12 healthy volunteers (HV), matched for age and sex with the two patient groups, respectively:

i. To identify the NP tract (HV-tractography group, aged 76.1 ± 1.5 years, 5 males, 7 females) by using tractography or
ii. To be used as a control group (HV-controls, aged 75.4 ± 1.6, 6 males, 6 females) in the comparison of NP diffusion metrics between AD and DLB subjects as described below.

All controls, recruited from the non-consanguineous relatives of the AD and DLB patients, were free from relevant pathologies and presented with a normal neurological examination, brief cognitive examination and brain MRI scan. Healthy volunteers did not undergo ^123^I-FP-CIT-SPECT imaging for radioprotection concerns and given the availability of published multicentre normative data for ^123^I-FP-CIT-SPECT [12].

### MRI protocol

MRI was performed with a 1.5-T scanner (Signa HDxt; GE Healthcare, Milwaukee, WI, USA) with an eight-channel transmit-receive head coil. The protocol included diffusion data and a volumetric T1-weighted sequence (coronal three-dimensional spoiled gradient recalled sequence, with repetition time 10.0 ms, echo time 4.5 ms, flip angle 8°, field of view 24 × 24 cm^2^ and matrix 256 × 256).

Diffusion-weighted imaging (DWI) was performed using an axial single-shot spin-echo echo-planar imaging sequence (repetition time 13,700 ms, echo time 90 ms, flip angle 90°, field of view 24 × 24 cm^2^ and matrix 96 × 96), with diffusion gradients applied in 61 non-collinear directions (*b* value = 1,000 s/mm^2^) and six *b* = 0 images for the HV-tractography group. The aim of this sequence was to identify the NP bundle in Montreal Neurological Institute (MNI) space as described below. All other subjects (AD, DLB, HV-controls) were scanned using a DWI sequence with 15 non-collinear directions (*b* value = 1,000 s/mm^2^) and two *b* = 0 images. Thus, the presence of any differences in diffusion metrics between HV-controls and patients were assessed using images acquired with the same sequence. While all images were collected on the same scanner, the differences in the DWI protocol between the HV-tractography group and all other subjects are due to the fact that the images for the DLB and AD patients were acquired as part of clinical work-up MRI exam. However, all DLB and AD subjects and all HV-controls subjects underwent the same diffusion MRI protocol with 15 directions described above.

### ^123^I-FP-CIT SPECT acquisition and analysis

For all AD and DLB subjects, ^123^I-FP-CIT SPECT data was acquired by means of a two-headed Millennium VG camera (General Electric Healthcare, Waukesha, WI, USA) and analysed using the BasGan algorithm [15]. Acquisition started between 180 and 240 min after injection of ^123^I-FP-CIT and lasted 40 min. A “step-and-shoot” protocol was applied with a radius of rotation < 15 cm, and 120 projections evenly spaced over 360° were generated. Total counts ranged between 2.5 and 3 million. The pixel size of the acquisition matrix was 2.4 mm, thanks to an electronic zoom (zoom factor 1.8) applied in the data collection phase. In the reconstruction phase, also a digital zoom was used and the resulting images were sampled by isotropic voxels with 2.33 mm sides. Projections were processed by means of the ordered subsets expectation maximisation (OSEM) algorithm (8 iterations, 10 subsets) followed by post filtering (three-dimensional Gaussian filter with full width-half maximum = 8 mm). The OSEM algorithm included a proback pair accounting for collimator blur and photon attenuation. No compensation for scatter was performed. The two-dimensional + 1 approximation was applied in the simulation of the space-variant collimator blur, whereas photon attenuation was modelled with the approximation of a linear coefficient uniform inside the skull and equal to 0.11 cm^−1^. The reconstructed ^123^I-FP-CIT SPECT images were exported in analysed format and processed by the automatic BasGan algorithm version 2 based on a high definition, three-dimensional striatal template, derived from Talairach’s atlas [16, 17]. Using this approach, an optimisation protocol automatically performs fine adjustments in the positioning of blurred templates to best match the radioactive counts and locates an occipital region of interest for background evaluation. Partial volume effect correction is included in the process of uptake computation of caudate, putamen and background. The partial volume effect correction performed by the method consists of an activity assignment in a Talairach-Tornoux atlas-based three-compartment model of basal ganglia. Background uptake was subtracted by putamen and caudate uptake as follows (caudate or putamen uptake − background uptake)/background uptake, to generate specific binding ratio (SBR) values. For this study, our metrics of interest were the putaminal SBR values.

### Tractography

Data were analysed using FMRIB Software Library (FSL) (http://fsl.fmrib.ox.ac.uk/fsl/fslwiki/) and MRtrix (http://www.mrtrix.org) packages. For each subject of the HV-tractography group, we used constrained spherical deconvolution (CSD) tractography to identify the NP.

**Fig. 1:**
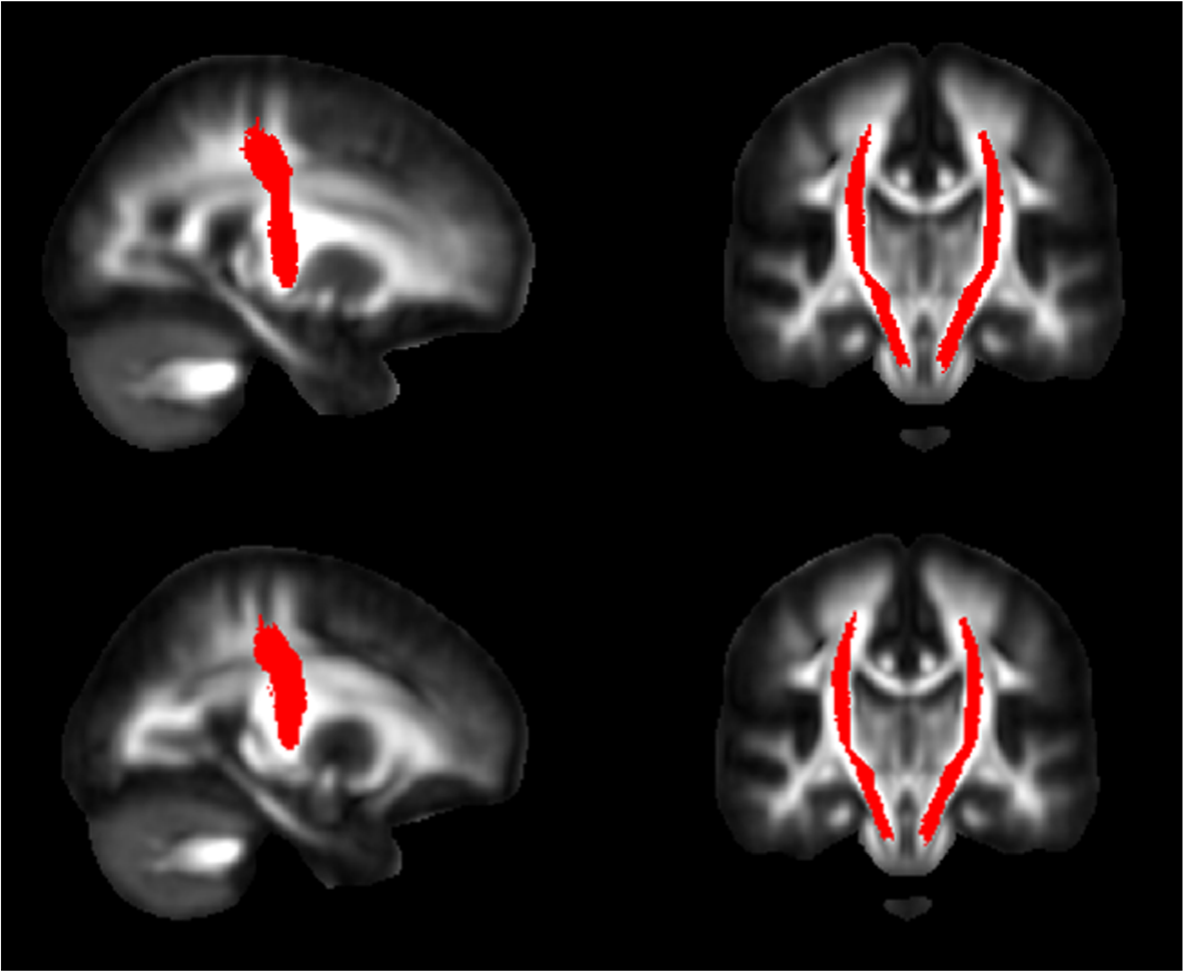
Bilateral corticospinal tract mask (in red) used as a control region

Our pipeline for nigroputaminal tractography was as follows:

i. *Generation of track density imaging (TDI) maps*. FSL [18] was used to perform eddy current corrections and brain extraction. Whole brain tractography was performed with MRtrix using an algorithm that combines CSD tractography with probabilistic tractography [19]. The relevant parameters were seed = whole brain, step-size = 0.1 mm, maximum angle between steps = 10°, maximum harmonics order = 8, and termination criteria: exit the brain or when the CSD fibre-orientation distribution amplitude was < 0.1 and generation of 2.5 million tracks. TDI map was created as the total number of tracks passing within each element of a user-defined super-resolution grid; for this study, a 1-mm grid was used. TDI maps have been shown to improve tractography results allowing a more precise visualisation of white matter tracts and tractography seed regions. TDI, for example, has been used to visualise brainstem nuclei (such as the red nucleus) [20], used for fibre tractography. In this study, TDI was used to verify the correct positioning of substantia nigra (SN) masks and to map tractography results in each individual subject native diffusion space.
ii. *Creation of SN and putamen nuclei masks.* Masks for the left and right SN and putamen nuclei were generated in MNI space using the WFU atlas toolbox included in statistical parametric mapping (SPM) (http://www.fil.ion.ucl.ac.uk/spm/). All the grey matter masks were registered from MNI space to each subject TDI maps using the nifty-reg software package [21]. To reach this aim, we co-registered each T1-weighted image to each subject native diffusion space, via the non-diffusion-weighted scans, using a linear transformation. We then used a non-linear transformation to co-register the MNI template brain to the native T1-weighted images, and then we concatenated the two transformations. The resulting transformation was then used to co-register the SN and putamen masks to each subject TDI image. The correct registration of each SN and putamen nuclei mask was then evaluated on TDI images and modified as needed to fit individual anatomy by a neurologist with more than 10 years of experience in neuroimaging and separately evaluated by a neuroradiologist with more than 15 years of experience in neuroimaging.
iii. *NP tractography*. It was performed in MRtrix with an algorithm which combines CSD with probabilistic streamline tractography (parameters: step-size = 0.1 mm, maximum angle between steps = 10°, maximum harmonics order = 8, and termination criteria: CSD fibre-orientation distribution amplitude was < 0.1, number of tracks: 3,000). Tractography algorithms, performed separately for the left and right NP tract used the TDI-registered SN and putamen nucleus masks as seed and target points. Resulting tracts were then mapped back to TDI images.
iv. *NP mask population mask.* The resulting tracts were translated into 1 × 1 × 1 MNI space using the non-linear registration function of the nifty-reg software package [21]. To reduce the possibility of including false-positive tracings, given the probabilistic nature of fibre tractography, but to allow for the natural variability of such a small pathway, we included in our final NP masks only those fibres’ reliability traced in at least 50% of healthy controls.
v. *Registration of NP masks to native diffusion space and extraction of FA and mean diffusivity values*. Nifty-reg was then used for each subject in the DLB, AD and HV-controls groups. The bilateral corticospinal tract (CST) mask, included in FSL, was used as a control region (Fig. 1). Regarding the registration of the NP bundle mask to each patient, two separate sets of analyses were performed, one using a non-linear registration approach (main results) and another using only a linear registration (confirmatory results).

### Statistical analysis

Statistical analysis was performed with SPSS version 23 (IBM; version 23.0). As preliminary analysis, a Shapiro-Wilk test was used to confirm the normality of the data. We then used both an ANOVA and an ANCOVA corrected for age and sex to evaluate group differences, parametric correlations to correlate DWI metrics with ^123^I-FP-CIT SPECT results and to calculate the DLB *versus* AD receiver operating characteristic curve (ROC) based on the tractography results. All analyses were repeated after bootstrapping with 3,000 permutations.

## Results

### Differences in diffusion metrics

**Fig. 2:**
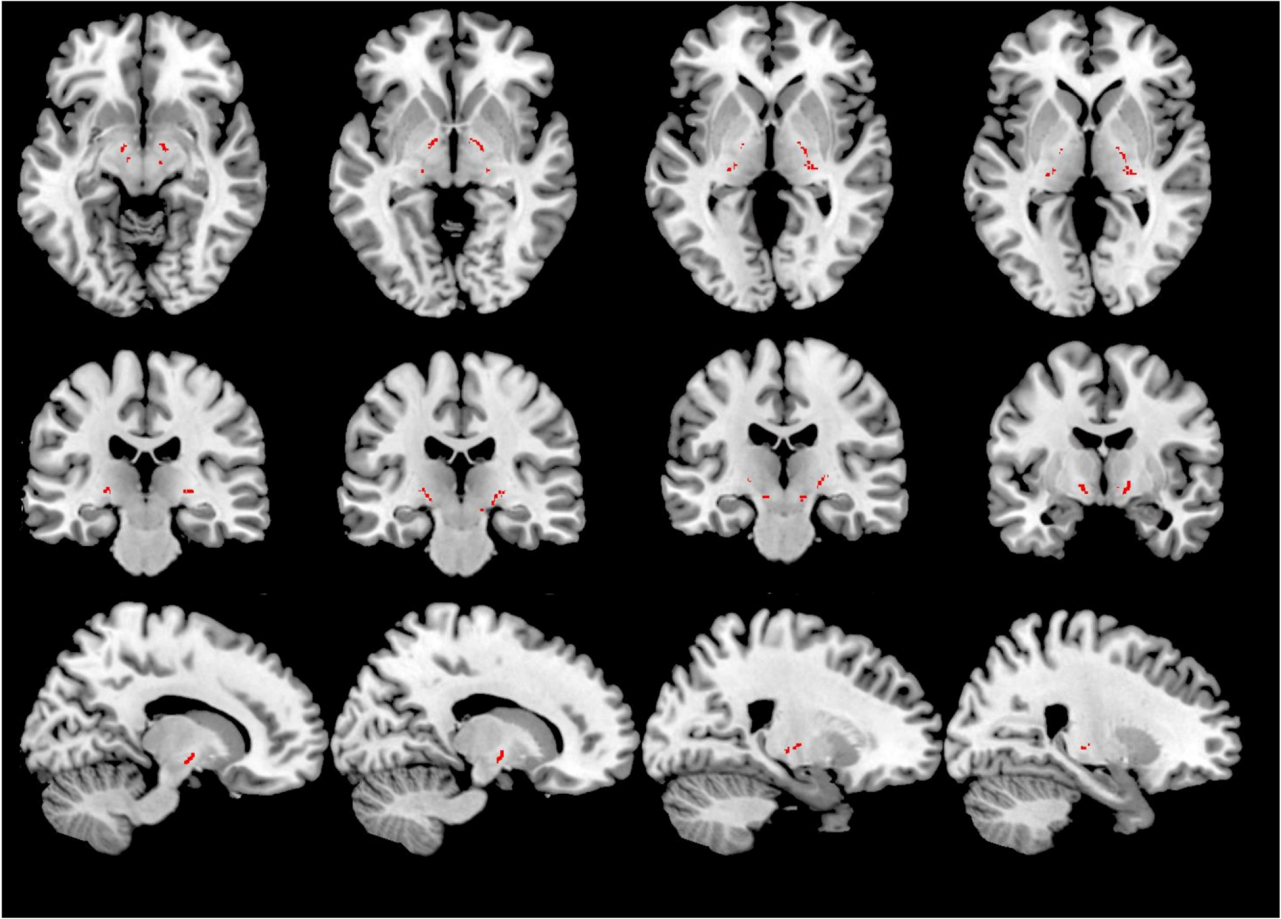
Left and right nigroputaminal tractography results (in red)

Demographic and clinical characteristics of AD, DLB and HV-controls are reported in Table 1. NP tractography results are shown in Fig. 2. NP FA values (average value of left and right NP tracts) were as follows: DLB, 0.500 ± 0.030; AD, 0.430 ± 0.040, and HV-controls, 0.420 ± 0.040 (Table 2). There was an overall group effect for FA (*F* = 14.1; *p* < 0.001) which remained significant correcting for age and sex (*p* = 0.002).

**Table 2:** Diffusion metrics (fractional anisotropy) values of nigroputaminal and corticospinal tracts across patients and controls

	**Alzheimer disease**	**Dementia with Lewy bodies**	**HV-controls**
**Nigroputaminal tract**	0.430 ± 0.040	0.500 ± 0.030	0.420 ± 0.040
**Corticospinal tract**	0.520 ± 0.040	0.530 ± 0.040	0.530 ± 0.030

Data are reported as mean ± standard deviation. *HV* Healthy volunteers

**Fig. 3:**
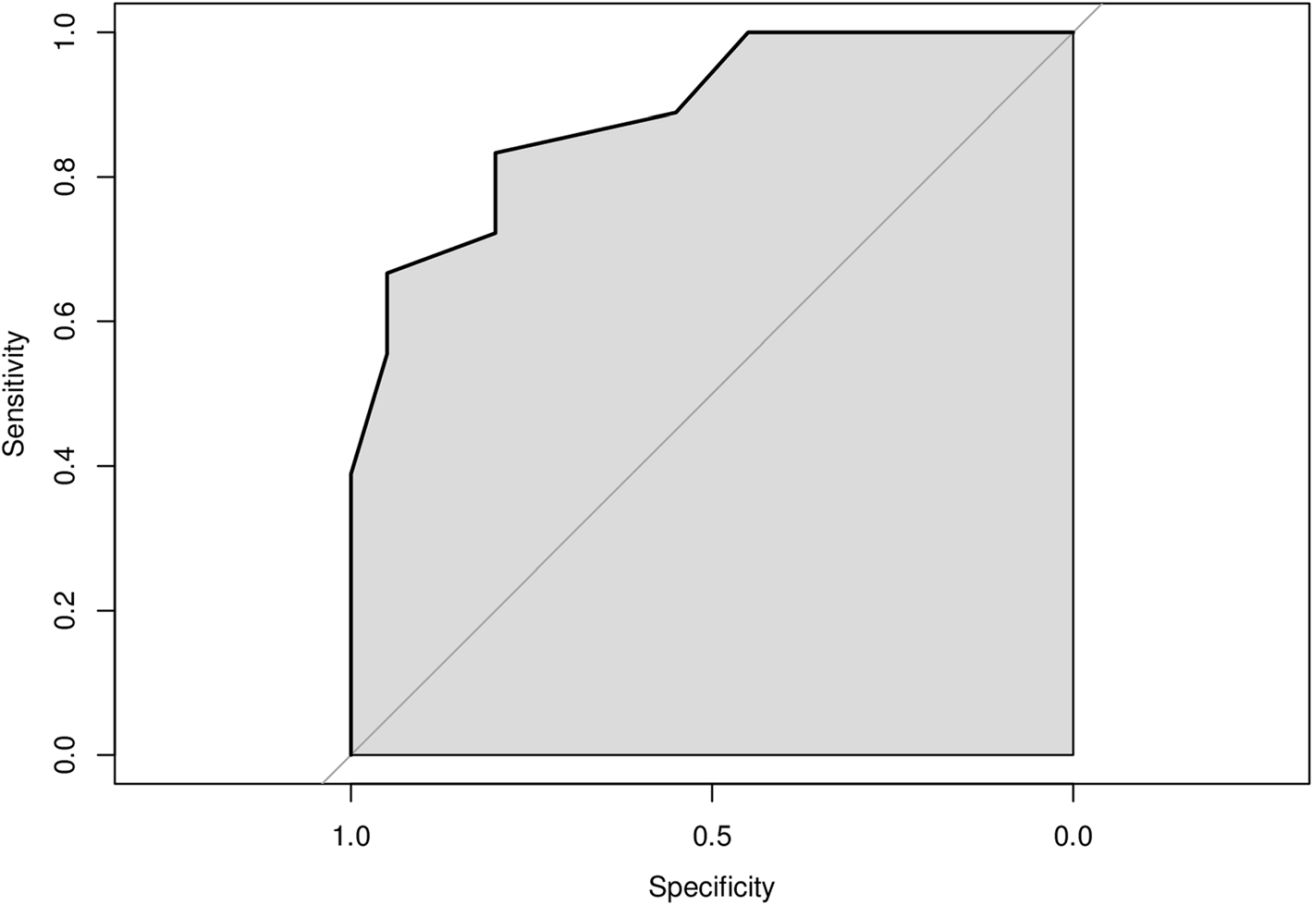
Receiver operating characteristic analysis for the discrimination between dementia with Lewy bodies and Alzheimer disease patients based on nigroputaminal fractional anisotropy values. The observed area under the curve value is 0.820

Patients in the DLB group presented with higher FA values compared with both AD and HV-controls groups (DLB vs HV-controls *p* = 0.001 [effect size *d* = 1.9, representing a large effect size]; DLB *versus* AD *p* = 0.001 [effect size *d* = 1.5, representing a large effect size]) in the NP masks, while there was no difference in NP FA between HV-controls and AD groups (*p* = 0.450). Based on these data, a ROC analysis was run; the area under the curve (AUC) for the discrimination of DLB and AD subjects based on NP FA values was 0.820 (Fig. 3).

Inside the CST mask, there was no significant difference in FA or MD values between the three groups (DLB 0.530 ± 0.040, AD 0.520 ± 0.040, HV-controls 0.530 ± 0.030) (Table 2). There was no significant group effect for NP or CST MD values (*p* = 0.740).

### Correlation between NP diffusion metrics and ^123^I-FP-CIT SPECT values

**Fig. 4:**
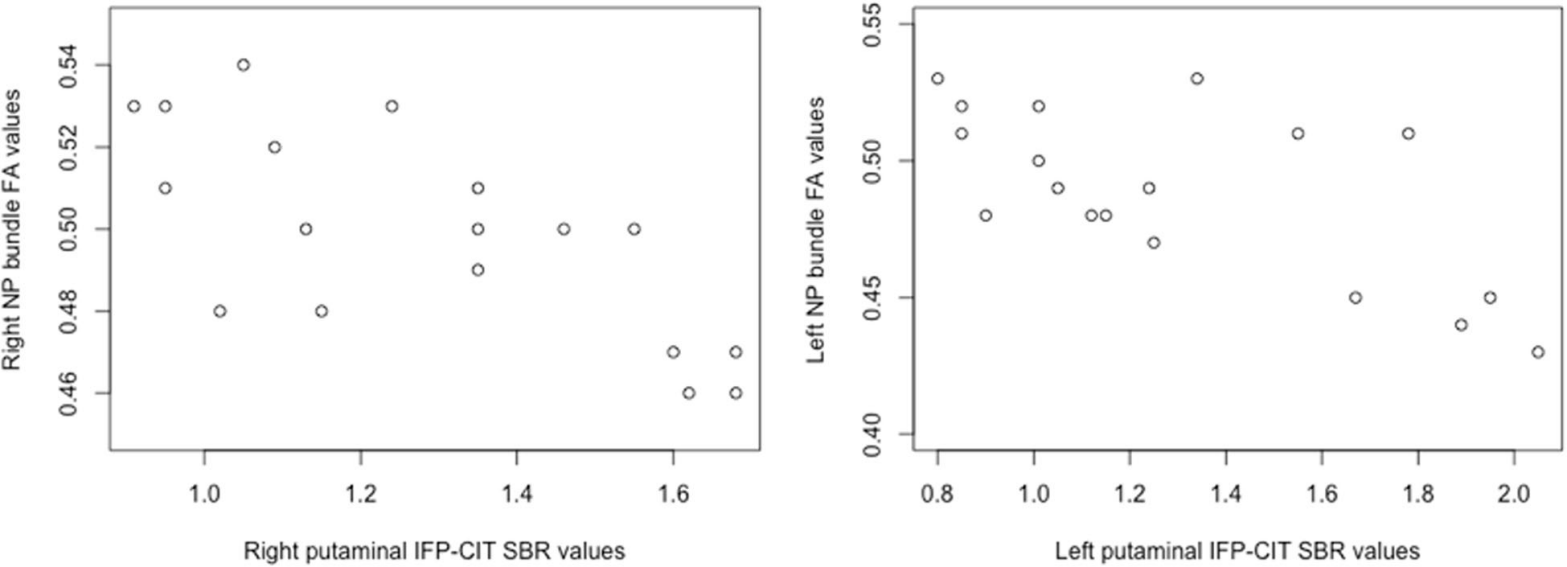
Correlations between putaminal ^123^IFP-CIT specific binding ratio (SBR) values and nigroputaminal (NP) fractional anisotropy (FA) in patients with dementia with Lewy bodies. Note the significant (right, *r* = -0.720, *p* = 0.001; left, *r* = -0.670, *p* = 0.003) inverse correlation between putaminal SBR values at ^123^IFP-CIT

Putaminal ^123^I-FP-CIT SPECT SBR values were 1.300 ± 0.410 and 1.260 ± 0.310 for the left and right putamen in the DLB group and 3.300 ± 0.890 and 3.000 ± 0.610 for the AD group. As per protocol, all DLB subjects and none of the AD subjects presented with ^123^I-FP-CIT SPECT SBR values suggestive of NP degeneration based on multicentre normative data [11]. Focusing on the DLB group, there was a significant inverse correlation between ^123^I-FP-CIT SPECT putamen SBR values and NP FA values (left, *r* = -0.670, *p* = 0.003; right, *r* = -0.720, *p* = 0.001; Fig. 4). There was no correlation between FA values inside the CST mask and ^123^I-FP-CIT SPECT putamen SBR values (left, *p* = 0.560; right, *p* = 0.450).

### Supplementary results

All analyses were re-run using a linear transformation to co-register the NP mask to each subject native diffusion space. There was no material difference in the results compared to those reported in the main analysis. NP FA values (average value of left and right NP tracts) were DLB 0.510 ± 0.030 (DLB), 0.440 ± 0.030 (AD) and 0.430 ± 0.040 (HV).

## Discussion

Here, we showed that diffusion-weighted MRI captures NP microstructural changes in DLB patients and thus could represent a useful approach to help in the differential diagnosis between DLB and AD. The finding of increased NP FA values in the DLB group should be interpreted in the context of two key anatomical features of the NP pathway, *i.e*, the presence of unmyelinated dopaminergic fibres (and thus their low FA values) and the presence of crossing fibres along its trajectory [22, 23] as well as the relative selective loss of NP fibres in DLB compared to AD.

In Huntington disease patients [22], selective loss of cortico-striato-thalamo-cortical fibres has been shown to lead to an FA increase in fronto-striatal white matter regions rich in crossing fibres. Similar regional findings of increased FA values in crossing fibre regions due to white matter fibre loss were also observed in multiple sclerosis [24] and in ageing [23]. In voxels rich in crossing fibres, the selective reduction of one of the fibre populations (and thus reduction of fibre dispersion) could lead to an increase in intravoxel FA, as observed in our study. This interpretation is in line with the key methodological facet of this study, *i.e.*, the use of a separate population of healthy controls to create a map of the NP fibre trajectory as opposed to identify the NP tract for each patient individually. Obviously, the mask-based approach provides information about the NP tracts’ macrostructural anatomy that are representatives of the mean values of the entire cohort of healthy subjects and not of DLB patients at an individual level. While this approach is free from the effect on probabilistic tractography of regional damage, it does not completely control (despite the use of a non-linear registration approach) for differences in regional atrophy [25]. Indeed, tractography analysis performed on images obtained by DLB patients may be affected by the structural degeneration of the NP tracts, thus reducing its reliability [26]. Future studies are needed to integrate the simple mask-based approach with a more detailed characterisation of the nigrostriatal system, based on graph-theory and including all the relevant grey matter regions and the connecting tracts.

It is not straightforward to compare our results with published data on nigrostriatal tractography in subjects with PD, given the differences in the target study population, the inclusion of AD patients in this study and our mask-based analysis approach. Moreover, the results of nigrostriatal tractography in PD are heterogeneous as nigrostriatal FA values in PD have been reported to be increased [27], decreased [8] or similar [9] of those of controls, and the same inconsistency of results has been reported for other metrics such as fibre density.

The heterogeneous nature of PD (for example regarding the motor phenotype) could at least partly explain these differences [28]. Another possible explanation may rely on the different pathological stages (from Lewy bodies’ formation to the final neuronal and axonal loss) of the neurodegenerative process affecting the NP tract at the time of MRI microstructural analysis [29]. Furthermore, FA is not able to reflect the specific pathological changes of the NP pathway. For example, a decrease in FA values may be due to the neurite density reduction, to an increase in the dispersion of neurite orientation distribution or demyelination.

A recent study [9], in which neurite orientation dispersion and density imaging (NODDI) have been used to evaluate the intracellular volume fraction and the orientation dispersion index in a cohort of PD patients, demonstrated a decrease in neurite density in the distal part of NP tract. This finding, consistent with histopathological studies suggesting that the degeneration in PD begins from the presynaptic terminals and the distal axons, may further suggest that different microstructural changes in different parts of the NP pathway at different time points characterise the neurodegenerative process, thus partly explain the conflicting results reported in the literature.

Despite the proof-of-concept nature of the current study, we run an exploratory ROC analysis of the potential of tractography to be used to differentiate between DLB and AD, showing good AUC values. Caution, however, is needed in the interpretation these ROC data given the nature of the DLB and AD subjects included here: small groups, the use of ^123^I-FP-CIT results as inclusion criteria in the study, the exclusion of subjects with vascular lesions in the basal ganglia region and the availability of a long follow-up to confirm the baseline diagnoses.

A main limitation of this work is represented by the use of diffusion-weighted MRI acquired in clinical practice at 1.5 T. However, we feel that the use of a NP population mask, the lack of significant effect on the analysis by the registration protocol and the use of ^123^I-FP-CIT as reference allows us to be confident with the presented results. The combination of MRI and ^123^I-FP-CIT has been previously used in the literature to gather multi-modal information of neurodegeneration [23]. Indeed, ^123^IFP-CIT, a non-invasive imaging method, characterised by limited exposure to ionising radiation and high cost-effectiveness, is one among the indicative biomarkers for the diagnosis of DLB in the current diagnostic criteria [1]. Therefore, alternative imaging biomarkers of NP degeneration, such as tractography-derived metrics of microstructural damage cited above (NODDI), must be validated by using ^123^IFP-CIT as reference standard comparator. Thus, in this context, our findings suggest a possible approach to NP degeneration assessment and point to the usefulness of a combined MRI/molecular imaging approach to validate tractography results. To increase our confidence with the baseline clinical diagnosis, we included only those subjects with at least 3 years of clinical follow-up thus also explaining why MRI images were not acquired using an up-to-date clinical scanner.

Further investigations performed on larger samples and based on higher field and standardised MRI protocols and processing analysis are necessary to confirm our findings.

## Data Availability

The dataset used and/or analysed during the current study is available from the corresponding author on reasonable request.

## Abbreviations

^123^IFP-CIT: Ioflupane
AD: Alzheimer disease
CSD: Constrained spherical deconvolution
CST: Corticospinal tract
DLB: Dementia with Lewy bodies
FA: Fractional anisotropy
HV: Healthy volunteers
NODDI: Neurite orientation dispersion and density imaging
NP: Nigroputaminal
OSEM: Ordered subsets expectation maximisation algorithm
PD: Parkinson’s disease
SBR: Specific binding ratio
SN: Substantia nigra
SPECT: Single-photon emission computed tomography
TDI: Track density imaging
